# Modulation of Cytokine-Induced Astrocytic Endothelin-1 Production as a Possible New Approach to the Treatment of Multiple Sclerosis

**DOI:** 10.3389/fphar.2019.01491

**Published:** 2020-01-08

**Authors:** Stéphanie Hostenbach, Miguel D’Haeseleer, Ron Kooijman, Jacques De Keyser

**Affiliations:** ^1^ Department of Neurology, Universitair Ziekenhuis Brussel, Vrije Universiteit Brussel (VUB), Brussels, Belgium; ^2^ Center for Neurosciences, Vrije Universiteit Brussel (VUB), Brussels, Belgium; ^3^ Department of Neurology, National Multiple Sclerosis Centrum, Melsbroek, Belgium; ^4^ Department of Neurology, Universitair Medisch Centrum Groningen (UMCG), University of Groningen, Groningen, Netherlands

**Keywords:** multiple sclerosis, endothelin-1, astrocytes, cytokines, inflammation, fluoxetine, simvastatin, resveratrol

## Abstract

**Background:** In the human central nervous system (CN), resting astrocytes do not visually show endothelin-1 (ET-1)-like immunoreactivity. In patients with multiple sclerosis (MS), an inflammatory disorder of the CNS, high levels of ET-1 are found in reactive astrocytes in demyelinated plaques. ET-1 may contribute to the pathology of MS by interrupting the blood-brain-barrier, enhancing inflammatory responses, excitotoxicity and reducing cerebral blood flow.

**Methods:** We used the human astrocytoma cell line 1321N1 to investigate the role of inflammatory cytokines involved in MS lesions (IL-1β, TNF-α, IFN-γ, LPS, IL-10, TGF-β) on astrocytic ET-1 upregulation. Prucalopride, rolipram, fenofibrate, fluoxetine, simvastatin, daglutril, and resveratrol were investigated as potential candidate drugs to suppress cytokine-induced astrocytic ET-1 production. Effects on ET-1 production were measured using both ELISA and RT-qPCR.

**Results and Conclusions:** ET-1 secretion by astrocytoma cells was only stimulated by the pro-inflammatory cytokines IL-1β and TNF-α. Fluoxetine, simvastatin, and resveratrol significantly inhibited this IL-1β- and TNF-α-induced ET-1 production. Simvastatin and resveratrol significantly reduced ET-1 mRNA levels, indicating an effect at the level of transcription. Fluoxetine significantly reduced endothelin converting enzyme-1 mRNA levels, suggesting and effect at the level of protein-processing. The required concentrations of simvastatin (>0.1 µM) and resveratrol (>10 µM) cannot be achieved in humans using pharmacologically accepted doses. Fluoxetine exerted a significant inhibitory effect on ET-1 secretion at a concentration of 5 µM, which is pharmacologically achievable in human brain, but the effect was modest (<50% suppression) and probably not sufficient to obtain a clinically relevant ET-1 effect. Our *in vitro* model can be a useful screening tool in the development of new drugs to suppress astrocytic ET-1 production. The effect of simvastatin was for the most part mediated *via* the mevalonate pathway, suggesting that this might be an interesting target for further drug development.

## Introduction

Multiple sclerosis (MS) is a chronic disorder of the central nervous system (CNS) that is pathologically characterized by the appearance of focal inflammatory lesions associated with demyelination and gliosis (plaques), disseminated in place and time. In addition, degenerative processes take place, including a progressive diffuse axonal degeneration and hippocampal neuronal loss (Compston and Coles, 2008). Destructive immune responses play a key role in the generation of focal lesions. The mechanisms responsible for the degenerative processes, which largely determine long-term disability in patients with MS are less well understood.

High levels of endothelin-1 (ET-1) have been found both in plasma and cerebrospinal fluid (CSF) of MS patients (Speciale et al., 2000; Haufschild et al., 2001). The likely source of this ET-1 production are reactive astrocytes in focal MS lesions, which express high levels of ET-1, while resting astrocytes in human brain visually do not show ET-1 immunoreactivity (D’Haeseleer et al., 2013). Mice with astrocytic ET-1 overexpression developed more severe experimental allergic encephalomyelitis (EAE), which is an animal model for the inflammatory lesions in MS (Guo et al., 2014).

The increased levels of ET-1 produced by reactive astrocytes may contribute to the pathology of MS by interrupting the blood-brain-barrier (BBB), enhancing the inflammatory responses, promoting excitotoxicity, and lowering cerebral blood flow (CBF) (Hostenbach et al., 2016). The manner by which astrocytes affect myelination seems to correlate with their level of reactivity (Nash et al., 2011; Kiray et al., 2016), and ET-1 released from reactive astrocytes acts as negative regulator of the differentiation of oligodendrocyte progenitor cells and remyelination (Hammond et al., 2014). ET-1 is also a potent vasoconstrictor, and previous research has shown that CBF in MS patients is already globally impaired from the early stages of the disease (Law et al., 2004; D’haeseleer et al., 2011).

Chronic cerebral hypoperfusion on itself may contribute to the pathology of MS. Animals subjected to chronic cerebral hypoperfusion developed axonal degeneration, focal white matter lesions with apoptosis of oligodendrocytes, myelin breakdown, inflammatory reactions, gliosis (Tomimoto et al., 2003), and neuronal loss in the hippocampal CA1 region (Ohta et al., 1997), which are all pathological features of MS.

We have shown that the oral administration of a single dose of the ET antagonist bosentan in MS patients can restore their CBF to values found in healthy volunteers (D’Haeseleer et al., 2013). This finding formed the basis for this study. The aim was to test a number of cytokines, found in MS lesions, for their ability to induce ET-1 production in astrocytic cells *in vitro*. The most relevant cytokine-induced *in vitro* model will then be used to screen a series of existing compounds for human use that pass the blood-brain barrier and may have potential to suppress ET-1 production.

The synthesis of ET-1 is mainly regulated at the transcription and translation level resulting in a 212-amino acid protein, preproET-1, which is further processed by a furin-like proprotein convertase to an inactive intermediate, big ET-1, which is then cleaved by an endothelin-converting enzyme (ECE) or other proteases into ET-1 (Hostenbach et al., 2016).

A number of drugs have been shown to influence ET-1 synthesis in other cell lines by acting at different levels of ET-1 expression. Others may on mechanistic grounds be candidate drugs to inhibit cellular ET-1 synthesis. For our study, the following compounds were selected: simvastatin, resveratrol, fluoxetine, prucalopride, rolipram, fenofibrate, and daglutril.

Simvastatin has been shown to downregulate ET-1 expression in human fetal astrocytes transfected with HIV-Tat protein, and decrease the transcription rate of the *ET-1* gene in bovine endothelial cells (Hernandez-Perera et al., 2000; Chauhan et al., 2007).

Resveratrol inhibited ET-1 mRNA expression in cultured endothelial cells through attenuating the activator protein-1 binding site (AP-1) of the ET-1 promotor (Liu et al., 2003).

Fluoxetine activates protein kinase A (PKA) in astrocytes and the ET-1 promotor element FoxO1 is a physiological substrate for PKA by the mean of phosphorylation and thus inhibition of FoxO1 (Lee et al., 2011).

Prucalopride reduced interferon-γ-induced MHC class II and B7 costimulatory immunostaining in cultured astrocytes. Furthermore, the drug is known to enhance the intracellular cAMP production, which in turn can activate PKA (Zeinstra et al., 2006).

Rolipram is an inhibitor of cyclic nucleotide phosphodiesterase responsible for the inhibition of the degradation of cAMP, which in turn will activate PKA. The drug has been shown to prevent ET-1 induced actions in perfused lung tissue of rat (Held et al., 1997).

Fenofibrate inhibits ET-1 expression in human endothelial cells, through enhanced expression of the transcriptional Küppel-like factor 11 which inhibits the ET-1 promotor, and on the other hand through inhibition of glycogen synthase kinase-3 activity, which will also inhibit ET-1 expression.

Daglutril has an endopeptidase (endothelin-converting enzyme) inhibiting effect and was shown to antagonize ET-1 induced vasoconstrictor activity in isolated human vaginal tissue (Rahardjo et al., 2013).

## Materials and Methods

### Regulation of ET-1 Production in Cultured Human Astrocytoma Cells

#### Astrocytoma Cell Line

The human astrocytoma cell line 1321N1 (gift from dr. Sarah Gerlo, Lab of Eukaryotic Gene Expression and Signal Transduction, Gent University, Belgium) was cultured in DMEM (Dulbecco’s Modified Eagle’s medium; Thermo Fisher, Belgium) with 10% FBS (Fetal Bovine Serum; Thermo Fisher, Belgium), 1% Fungizone (Thermo Fisher Belgium), and 1% Pen-strep (Penicillin-Streptomycin-medium; Thermo Fisher Belgium) in a humidified 5% CO_2_ atmosphere at 37°C. After approximately 1 week, they were fully grown and plated out in 12-well plates at a concentration of 30,000 cells per 2 ml DMEM. After 3 days, cells were confluent and used for the experiments described below.

#### Incubation With Inflammatory Cytokines

Cells were cultured for 6 h in either the absence or presence of inflammatory modulators, after which the supernatant was collected for the measurement of ET-1 and frozen at -80°C. A number of pro-inflammatory and anti-inflammatory cytokines were tested: TNF-α (Tumor Necrosis Factor alfa; Miltenyi Biotec, The Netherlands) at concentrations of 1, 10, 50, 100, and 250 ng/ml, IFN-γ (Interferon gamma; Life Technologies, Belgium) at a concentration of 100 ng/ml, IL-1β (Interleukin -1 beta; Life Technologies, Belgium) at 1, 10, 50, 100, and 250 ng/ml, LPS (Lipopolysacharide; Sigma Aldrich, Germany) at 0.5 and 10 μg/ml, thrombin (Sigma Aldrich, Germany) at 3 units/ml, IL6 (interleukin 6; R&D systems, Germany) at 10 ng/ml and 100 ng/ml, IL-10 (interleukin-10; Life Technologies, Belgium) at 10ng/ml and 100ng/ml and TGF-β (Transforming Growth Factor -beta; Sigma Aldrich, Belgium) at 10 ng/ml and 100 ng/ml. TNF-α, IL-1β, IL6 and IL10 were solved in LPS-free water (pharmacy University Hospital Brussels); LPS in PBS; thrombin in Ultrapure Water (Sartorius Biotech, type: Arium^®^ Pro UV, Germany); IFN-γ in BSA (N,O-bis (trimethylsilyl) acetamide, Sigma Aldrich, Germany). The concentrations of the cytokines used were chosen according to previous experiments in cell cultures reported in the literature.

#### Incubation With Compounds to Suppress ET-1 Secretion

All drugs were dissolved in a proper dissolvent: prucalopride (Selleckchem, The Netherlands), rolipram (Tocris Bioscience, UK), fenofibrate (Sigma Aldrich, Germany), simvastatin (Merck Millipore, United Kingdom), daglutril SLV 306 (Axon Medchem, The Netherlands), resveratrol (Sigma Aldrich, Germany) in Dimethyl Sulfoxide (DMSO; Sigma Aldrich, Germany), and fluoxetine (Sigma Aldrich, Germany) in Ultrapure water (Sartorius Biotech, type: Arium^®^ Pro UV, Germany).

Different concentrations of all the components were tested starting from active concentrations used in the literature. Prucalopride at concentrations of 50nM, 250 nM, 500nM; rolipram at 1µM, 5µM, 10µM; fenofibrate at 10µM, 50µM, 100µM; simvastatin at 1nM, 10nM, 100nM, 5µM, 25µM; daglutril at 1µM, 10µM, 50µM; resveratrol at 1µM, 10µM, 100µM, and fluoxetine at 1 µM, 5µM, and 10µM.

In the course of the experiments we also tested dibutyryl-cAMP (dbcAMP, Sigma Aldrich, Germany) in Ultrapure water at concentrations of 100µM, 250µM, and 500µM and mevalonate (Sigma Aldrich, Germany) in DMSO at concentrations of 10 µM and 100 µM.

To evaluate the effects of the selected drugs on ET-1 secretion, astrocytoma cells were incubated with the compound or vehicle for 24 h before the addition of TNF-α and IL-1β at a final concentration of 100 ng/ml each and supernatant for ET-1 measurements was taken 6 h after their administration. We used the combination of both cytokines because both are present in MS lesions.

#### Enzyme-Linked Immunosorbent Assay (ELISA)

Concentrations of ET-1 in the supernatant of the cultured human astrocytoma cells were measured using the Endothelin Pan Specific ELISA kit^®^ (R&D systems, Abingdon, UK), according to the manufacturer’s instructions. This kit not only measures ET-1 but has a cross-reactivity with both ET-2 and ET-3. A study reported that neonatal rat astrocytes also produce ET-3 (Ehrenreich et al., 1991). We tested a specific Endothelin-1 ELISA kit (IBL International, Hamburg, Germany) and found that ET-1 concentrations were the same as the ET concentrations measured with the Pan Specific ELISA kit. Therefore, for the screening experiments we used the Endothelin Pan Specific ELISA kit^®^ to reduce the costs.

#### Quantitative Real-Time Polymerase Chain Reaction (RT-qPCR)

RNA was isolated from cell pellets of cultured human astrocytoma cell line using the RNeasy Mini Kit^®^ (Qiagen, Hilden, Germany), according to the manufacturer’s instructions. cDNA was reversely transcribed using TaqMan™ Reverse Transcription Reagents (Thermo Fisher Scientific, Belgium). The expression of the transcripts for ET-1, ECE-1, and GAPDH were assessed using *TaqMan*™ gene expression assays with respectively following assay IDs: Hs00174961_m1, Hs01043735_m1, Hs00206701_m,1 and Hs02758991_g1. The mRNA levels of ET-1 and ECE-1 were normalized to GAPDH mRNA expression.

### Statistical Analyses

Statistical analyses were performed using GraphPad Prisms 6.0b software. Data in all experiments are presented as the mean ± standard deviation (SD) of at least 4 independent experiments. Significant differences were tested with either the Mann Whitney U-test or the Kruskal-Wallis-test (including the Dunn’s Multiple Comparisons Test). Values were considered statistically significant when P < 0.05.

## Results

### Upregulation of ET-1 by Inflammatory Cytokines

**Figure 1:**
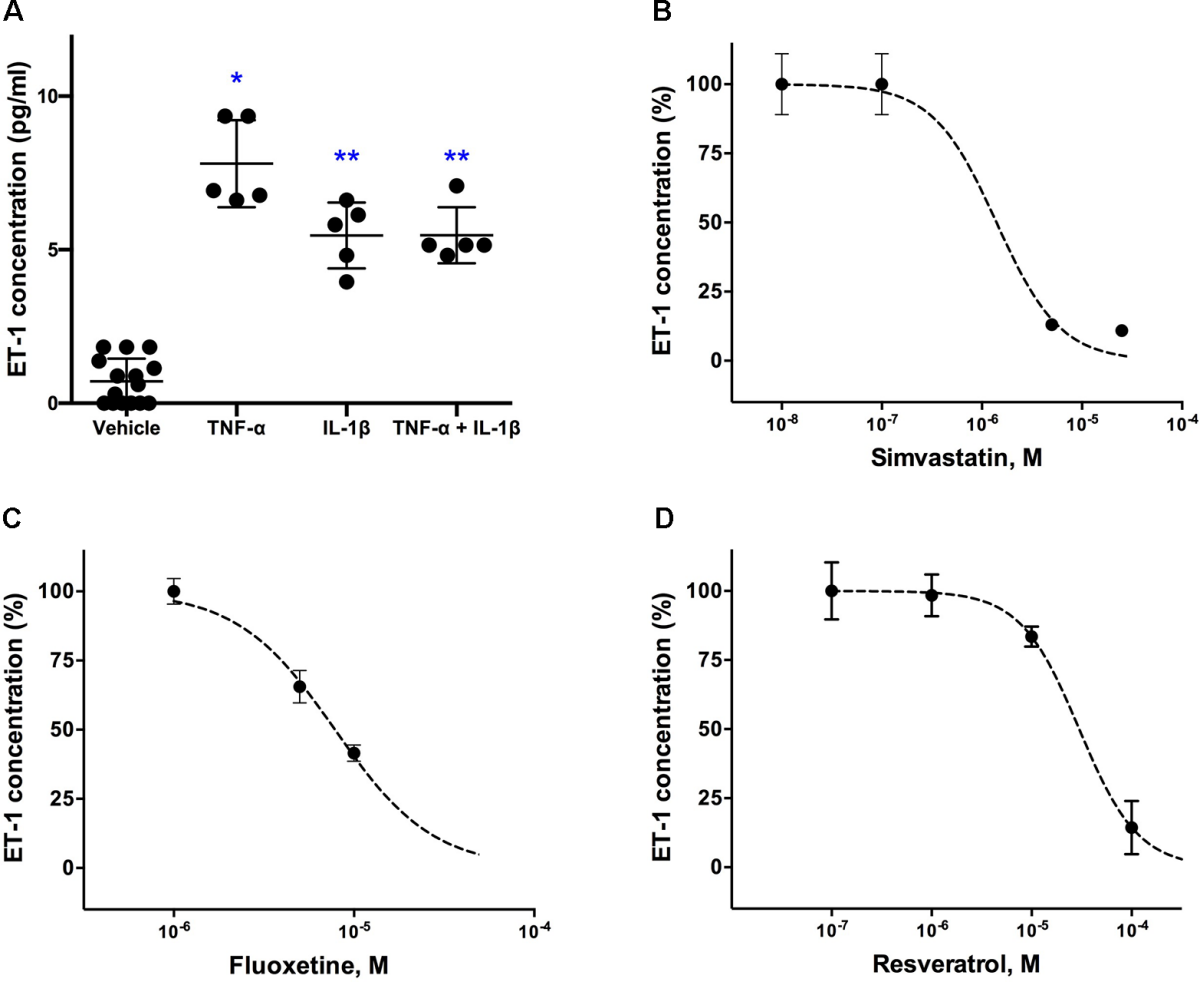
Effects of **(A)** pro-inflammatory cytokines (TNF-α and IL-1β) on ET-1 secretion in cultured human astrocytoma cells and inhibitory concentration-response curves for **(B)** simvastatin, **(C)** fluoxetine and **(D)** resveratrol. Cytokines were administrated to obtain a final concentration of 100 ng/ml each and supernatant for ET-1 measurements was taken 6 h after their administration (n = 5). For the inhibitory experiments (n = 4), cells were pre-incubated with the drug for 24 h before their stimulation with the inflammatory cytokines. Data are presented as means ± SD. Dose-response curves were generated with GraphPad Prisms 6.0b software. ^∗^ *P* < 0.001 and ^∗∗^ *P* < 0.05 vs. the vehicle group.

**Figure 1A** shows that cultured astrocytoma cells produce very low basal levels of ET-1 and that TNF-α and IL-1β, both at a concentration of 100ng/ml, significantly increased ET-1 levels in the culture medium. No further increase of ET-1 levels was obtained at concentrations of 250ng/ml. In the Kruskal-Wallis-test, the increase in ET-1 was not significantly different between TNF-α and either IL-1β or the combination of both cytokines.

Effects of the other tested cytokines (INFγ, LPS, thrombin, IL-6, IL-10, TGF-β) were not statistically significant (not shown).

### Effects of the Compounds on ET-1 Secretion

A concentration-dependent decrease in ET-1 secretion was found for simvastatin (**Figure 1B**), fluoxetine (**Figure 1C**), and resveratrol (**Figure 1D**). Simvastatin, fluoxetine, and resveratrol did not affect basal (noncytokine stimulated) ET-1 levels in the culture medium.

Incubations with prucalopride, rolipram, fenofibrate, and daglutril were without effect (not shown). Furthermore, in the course of the experiments with the drugs fluoxetine, prucalopride, and rolipram, we also tested the effect of dbcAMP on ET-1 secretion, since this component can activate PKA which in turn may regulate the ET-1 promoter element FoxO1. We tested dbcAMP in different concentrations, but there was no significant effect on ET-1 secretion (not shown). This indicates that drugs acting through the cAMP pathway have no effect on the ET-1 production, and that the positive effect of fluoxetine was likely obtained through a cAMP independent mechanism.

**Figure 2:**
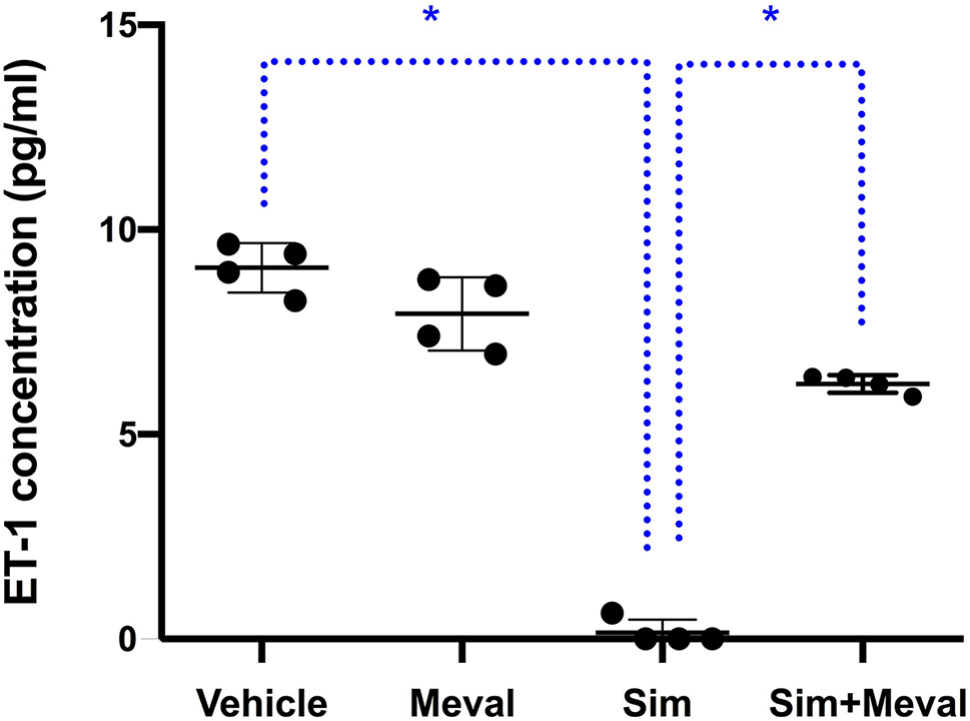
Effects of mevalonate (Meval) 10μM on the inhibiting effect of 5μM simvastatin (Sim) on ET-1 secretion in cultured astrocytoma cells. Cells were pre-incubated with the compounds for 24 h before their stimulation with TNF-α and IL-1β. Mevalonate alone had no effect on ET-1 secretion but significantly attenuated the inhibiting effect of simvastatin on ET-1 secretion (n = 4). Data are presented as means ± SD. *P* < 0.05 vs. simvastatin. *p = 0.0286.

To test whether suppression the cholesterol synthesis pathway is key to the inhibition of ET-1 secretion by simvastatin, we tested the effect of mevalonate supplementation in the presence of simvastatin (**Figure 2**). Addition of mevalonate 10µM to the cells for the most part attenuated the inhibiting effect of simvastatin on ET-1 secretion, indicating that simvastatin decreases ET-1 levels, at least partially, through the mevalonate-pathway. Mevalonate alone did not have a significant effect. A higher concentration of mevalonate (100µM) could not further attenuate the inhibiting effect of simvastatin (not shown).

**Figure 3:**
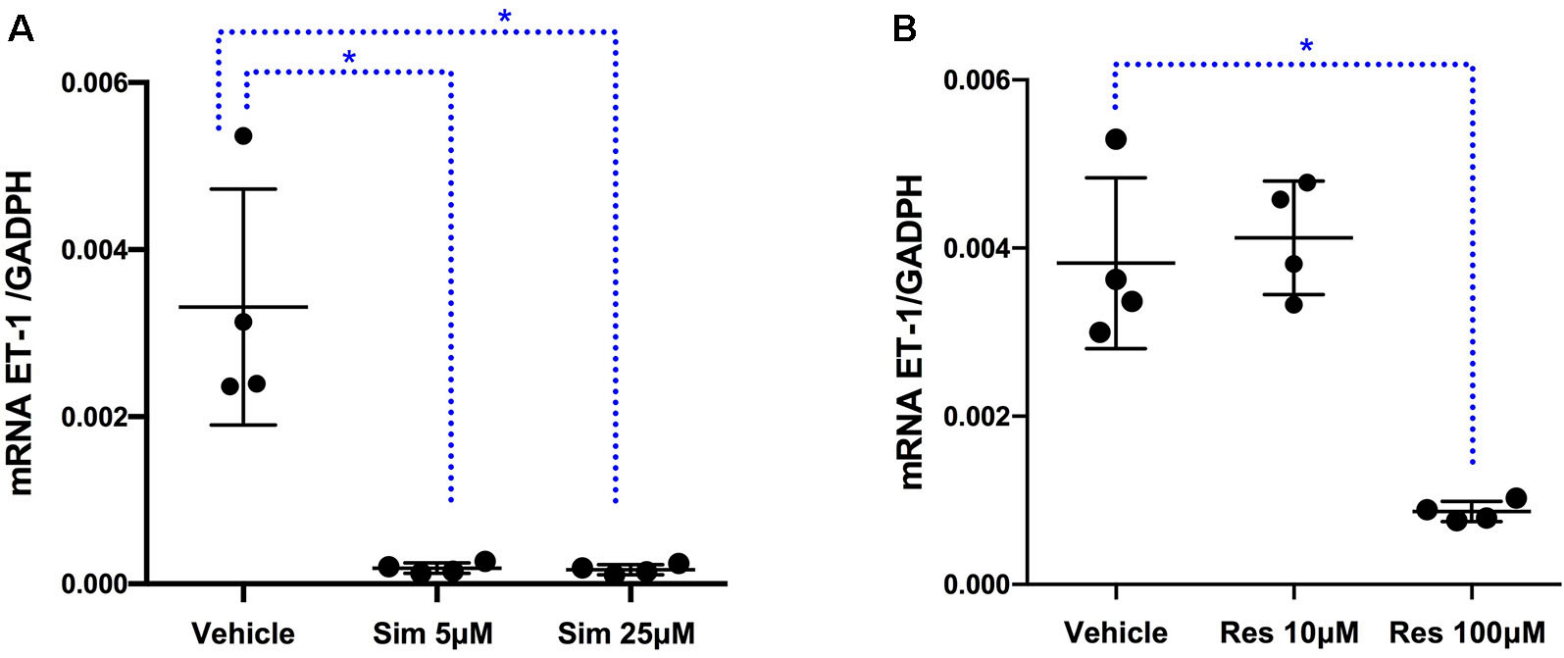
Effects of **(A)** simvastatin (^∗^ *P* < 0.05) and **(B)** resveratrol (^∗^ *P* < 0.05) on ET-1 mRNA expression. Results were normalized to housekeeping GADPH mRNA expression. Cells were pre-incubated with the compounds for 24 h before their stimulation with TNF-α and IL-1β. RNA was isolated from the human astrocytoma cell pellets taken 6 h after their administration. Data are presented as means ± SD (n = 4).

**Figure 4:**
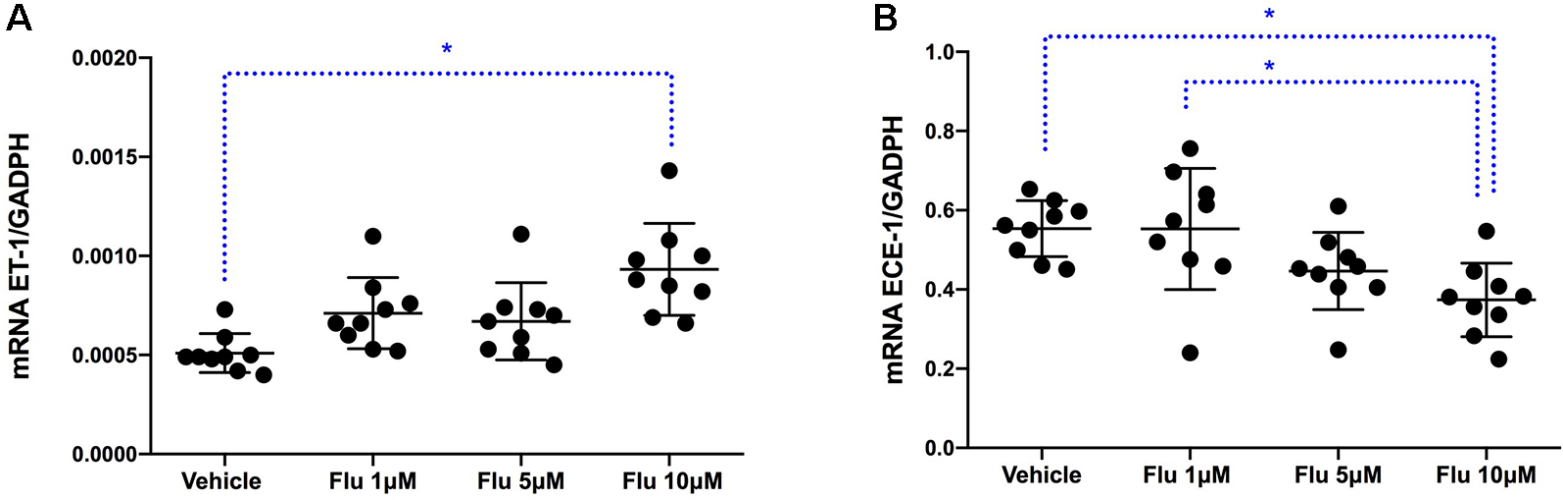
Effects of fluoxetine on **(A)** ET-1 mRNA expression (^∗^ *P* < 0.01) and **(B)** ECE-1 mRNA expression (^∗^ *P* < 0.01). Results were normalized to housekeeping GADPH mRNA expression. Cells were pre-incubated with the compounds for 24 h before their stimulation with TNF-α and IL-1β. RNA was isolated from the human astrocytoma cell pellets taken 6 h after their administration. Data are presented as means ± SD (n = 9).

**Figure 5:**
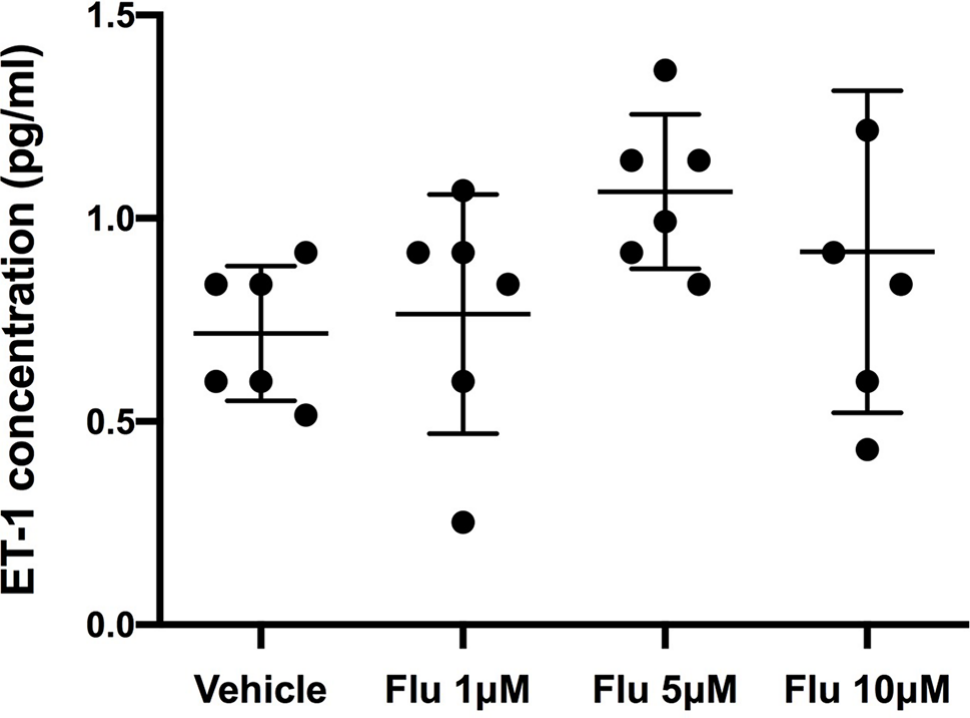
Intracellular levels of ET-1 after the administration of different concentration of fluoxetine (Flu). Cells were pre-incubated with fluoxetine for 24 h before their stimulation with TNF-α and IL-1β. Concentrations of ET-1 were measured in cell lysates taken 6 h after their administration. There were no significant differences between the intracellular ET-1 concentrations. Data are presented as means ± SD (n = 6).

To assess at which level the production of ET-1 is regulated by fluoxetine, simvastatin, and resveratrol, we measured ET-1 mRNA levels. Both simvastatin (5 and 25 μM) and resveratrol (100 μM) significantly decreased the levels of ET-1 mRNA, indicating that these drugs act at the level of transcription (**Figure 3**). In contrast, fluoxetine 5 and 10µM was associated with an increase in ET-1 mRNA levels and a decrease in ECE-1 mRNA levels (**Figure 4**). Intracellular protein levels were not affected by fluoxetine (**Figure 5**). Taken together, our findings suggest that fluoxetine decreases ET-1 production by reducing ECE-1, which converts big ET-1 to ET-1.

## Discussion

We found that ET-1 secretion in human astrocytoma cells was stimulated by the pro-inflammatory cytokines IL-1β and TNF-α, which are known to be present in focal MS lesions (Bittner et al., 2014). The ET-1 promotor contains response elements for activator protein-1 (AP-1), which is the most important regulator for ET-1 (Stow et al., 2011), FoxO1 (Nicholson et al., 2010; Lee et al., 2011), and NF-κB (Morishita et al., 2014). The presence of the NF-κB response element can explain the stimulating effect of these pro-inflammatory cytokines.

We found no statistically significant difference in the level of ET-1 production by the astrocytoma cells using the highest concentration of TNF-α and the highest concentration of IL-1β. There was no additive effect by using both cytokines, which may be explained by the fact that each cytokine on itself already produced a maximal effect on ET-1 production, and it suggests that both cytokines act through the same mechanism.

Among the compounds tested, only simvastatin, resveratrol and fluoxetine significantly inhibited ET-1 production in human astrocytoma cells. Release of ET-1 by reactive astrocytes can be regulated at different levels, including transcription, translation, protein-processing or secretion of ET-1. The suppressive effect on ET-1 production by simvastatin and resveratrol was regulated at the mRNA level, whereas fluoxetine, at least partially, acted at the level of protein-processing.

We found that concentrations of 5 and 25 µM of simvastatin were needed to suppress transcription of the *ET-1* gene and production of ET-1. With both concentrations a decrease of 89% of the ET-1 concentrations was obtained. However, these concentrations can never be reached in human brain when pharmacological doses of simvastatin between 20 mg and 80 mg (high dose simvastatin) are used. Pleiotropic effects of statins in previous *in vitro* cell experiments not related to ET-1 production were also found at concentrations of 1–50 µM (Björkhem-Bergman et al., 2011). However, the mean concentration of statins in human serum after therapeutic doses is 1000 times lower (1–15 nM). Furthermore, only 1%–5% of this quantity is pharmacologically active and only one third of this serum concentration can reach the CNS (Björkhem-Bergman et al., 2011). Our results demonstrated that the effect of simvastatin was at least for the most part mediated *via* the mevalonate pathway, suggesting that this might be an interesting target for further drug development.

Simvastatin has been investigated in clinical trials in patients with MS. A meta-analysis performed in 2012 concluded that the addition of statins to interferon therapy did not significantly influence the relapse risk, disease progression, or EDSS scores in patients with relapsing remitting MS (Bhardwaj et al., 2012). A small study presented in 2014 suggested that simvastatin 80 mg a day in patients with secondary progressive MS might reduce the rate of whole-brain atrophy compared with placebo (Chataway et al., 2014). A phase 3 trial to confirm this effect in secondary progressive MS is ongoing in the UK (Williams et al., 2019). If simvastatin would have a beneficial effect in MS, our study suggests that it is not due to an effect on ET-1 production.

Fluoxetine concentrations of 5 and 10 µM significantly reduced ET-1 secretion, corresponding with a concentration decrease of 31% and 45%, respectively. Treatment of humans for 29 days with 40 mg fluoxetine resulted in brain fluoxetine levels of approximately 5 µM as assessed with magnetic resonance spectroscopy (Karson et al., 1993). A dose of 40 mg fluoxetine, which is often used in clinical practice, may thus be able to reduce ET-1 concentrations in brain, but the effect is probably too small to obtain a clinically significant effect, where an almost complete suppression of ET-1 should be achieved. Our findings that prucalopride, rolipram, and dbcAMP had no influence on astrocytic ET-1 secretion argue against the possibility that the effect of fluoxetine was mediated through the cAMP-dependent PKA pathway.

It has been shown that oral administration of fluoxetine in mice prevented EAE or ameliorated ongoing EAE. This was associated with a downregulation of different inflammatory cytokines (Il-6, IL-10, TNF-α, among others), indicating that this was the result of immunomodulatory effects of fluoxetine (Bhat et al., 2017). Preliminary evidence of a possible immunomodulatory effect of fluoxetine was also found in a small pilot study in patients with relapsing remitting MS. A daily dose of 20 mg fluoxetine tended to reduce the formation of new inflammatory lesions on magnetic resonance imaging of the brain compared to placebo (Mostert et al., 2008). Two randomized placebo-controlled trials with a daily dose of 40 mg of fluoxetine in patients with progressive MS, which reflects progressive axonal degeneration that proceeds rather independently of inflammation, failed to show a neuroprotective benefit (Wood, 2018; Cambron et al., 2019).

Resveratrol, a dietary antioxidant polyphenol is present in a number of regularly consumed plant species like berries, grapes and peanuts and is a major constituent of red wine. In a study with healthy volunteers, a single dose of 25 mg resveratrol was given as a dietary supplement. The concentration of the ^14^C-labelled resveratrol measured with high-performance liquid chromatography, 1 h after oral intake, was very low in the systemic circulation (about 2 µM), due to a very rapid and extensive metabolism by the bacterial flora in the human intestine. In another study where 5 g of resveratrol was administered orally to ten healthy volunteers, the maximum plasma concentration reached was 2, 36 µM (Walle et al., 2004). None of the *in vivo* pharmacokinetic studies in humans have shown plasma concentrations greater than 10 µM. In the brain, the concentration will probably be even lower. Resveratrol was well-tolerated and adverse reactions were mild at a dose of maximally 1 g a day; above this dose diarrhea was frequently reported. This dose can be assumed as the upper limit for clinical trials. In our study, we only found an inhibitory effect of resveratrol on ET-1 production by the astrocytoma cells at a concentration of 100 µM, but not at 10 µM.

A limitation of our study is that we used a human astrocytoma cell line as screening model, because they differ from primary human astrocytes and do not completely reflect the *in vivo* situation. However, the human astrocytoma cell line that we used is a well-established stable cell line capable of responding to cytokine exposure in a manner typical of reactive astrogliosis and is therefore a valuable cellular model in the assessment of *in vitro* drug screening. It is probably more relevant to the human response than existing animal cell-based models. It was our intention to confirm clinically significant positive findings in cultured human astrocytes. However, we did not proceed further because all results in the screening phase with the human astrocytoma cell line were disappointing.

## Conclusion

Drugs that inhibit inflammation-induced ET-1 production in reactive astrocytes might widen the therapeutic arsenal in MS. Fluoxetine, simvastatin, and resveratrol, which are all drugs able to pass the blood-brain-barrier, suppressed inflammation-induced ET-1 secretion in cultured human astrocytoma cells. However, only fluoxetine exerted an effect at concentrations that are pharmacologically achievable in humans, but the effect was modest and probably insufficient to obtain a clinically relevant effect. Our *in vitro* model can be useful screening tool in the development of new drugs to suppress astrocytic ET-1 production, which must then be able to use in pharmacologically feasible doses. The mevalonate pathway might be an interesting target for further drug development. Suppressing astrocytic ET-1 production may be a potential therapeutic target in diverse other neurodegenerative disorders associated with reactive astrocytosis (Hostenbach et al., 2016). Due to the current lack of a suitable compound to suppress astrocytic ET-1 production we have started a phase 2 trial in MS patients with the ET-1 receptor antagonist bosentan (Hostenbach et al., 2019).

## Data Availability Statement 

The datasets generated for this study are available on request to the corresponding author.

## Author Contributions

SH, RK, and JK designed the experiments and interpreted the results. SH performed the experiments, analyzed the data, and wrote the manuscript. All authors discussed the results. MD’H, RK, and JK made critical revisions to the manuscript. All authors read and approved the final version of the manuscript.

## Funding

Research Foundation Flanders (FWO). SH is an FWO research fellow.

## Conflict of Interest

The authors declare that the research was conducted in the absence of any commercial or financial relationships that could be construed as a potential conflict of interest.
